# Recognition of prokaryotic and eukaryotic promoters using convolutional deep learning neural networks

**DOI:** 10.1371/journal.pone.0171410

**Published:** 2017-02-03

**Authors:** Ramzan Kh. Umarov, Victor V. Solovyev

**Affiliations:** 1 King Abdullah University of Science and Technology, Thuwal, Saudi Arabia; 2 Softberry Inc., Mount Kisco, United States of America; National Center for Biotechnology Information, UNITED STATES

## Abstract

Accurate computational identification of promoters remains a challenge as these key DNA regulatory regions have variable structures composed of functional motifs that provide gene-specific initiation of transcription. In this paper we utilize Convolutional Neural Networks (CNN) to analyze sequence characteristics of prokaryotic and eukaryotic promoters and build their predictive models. We trained a similar CNN architecture on promoters of five distant organisms: human, mouse, plant (*Arabidopsis*), and two bacteria (*Escherichia coli* and *Bacillus subtilis*). We found that CNN trained on sigma70 subclass of *Escherichia coli* promoter gives an excellent classification of promoters and non-promoter sequences (Sn = 0.90, Sp = 0.96, CC = 0.84). The *Bacillus subtilis* promoters identification CNN model achieves Sn = 0.91, Sp = 0.95, and CC = 0.86. For human, mouse and *Arabidopsis* promoters we employed CNNs for identification of two well-known promoter classes (TATA and non-TATA promoters). CNN models nicely recognize these complex functional regions. For human promoters Sn/Sp/CC accuracy of prediction reached 0.95/0.98/0,90 on TATA and 0.90/0.98/0.89 for non-TATA promoter sequences, respectively. For *Arabidopsis* we observed Sn/Sp/CC 0.95/0.97/0.91 (TATA) and 0.94/0.94/0.86 (non-TATA) promoters. Thus, the developed CNN models, implemented in CNNProm program, demonstrated the ability of deep learning approach to grasp complex promoter sequence characteristics and achieve significantly higher accuracy compared to the previously developed promoter prediction programs. We also propose random substitution procedure to discover positionally conserved promoter functional elements. As the suggested approach does not require knowledge of any specific promoter features, it can be easily extended to identify promoters and other complex functional regions in sequences of many other and especially newly sequenced genomes. The CNNProm program is available to run at web server http://www.softberry.com.

## Introduction

Promoter is a key region that is involved in differential transcription regulation of protein-coding and RNA genes. Gene-specific architecture of promoter sequences makes it extremely difficult to devise the general strategy for their computational identification [1, 2]. Promoter 5’-flanking regions may contain many short (5–10 bases long) motifs that serve as recognition sites for proteins providing initiation of transcription as well as specific regulation of gene expression.

A minimal eukaryotic promoter region, called core promoter, is capable of initiating basal transcription and contains a transcription start site (TSS). About 30–50% of all known eukaryotic promoters contain a TATA-box at a position ~30 bp upstream from the transcription start site. Many highly expressed genes contain a strong TATA box in their core promoter. At the same time, large groups of genes including housekeeping genes, some oncogenes and growth factor genes possess TATA-less promoters. In these promoters Inr (the initiator region) or the recently found downstream promoter element (DPE), usually located ~25–30 bp downstream of TSS, may control the exact position of the transcription start [1, 2].

Bacterial promoters contain two short conserved sequence elements approximately -10 and -35 nucleotides upstream from the transcription start site. The -10 box is absolutely essential to start transcription in prokaryotes. The sequence of -35 box affects the transcription rate [3–6]. Those consensus sequences, while conserved on average, are not found intact in most promoters.

Accurate prediction of promoters is fundamental for interpreting gene expression patterns, and for constructing and understanding genetic regulatory networks. In the last decade, genomes of many organisms have been sequenced and their gene content was mainly computationally identified. Promoters and transcriptional start sites (TSS), however, are still left largely undetermined and the efficient software able to accurately predict promoters in newly sequenced genomes is not yet available in public domain.

There were many attempts to develop promoter prediction software as for bacterial as well as for eukaryotic genomes. Most of them implemented very diverse computational algorithms, which often account some specific sequence features discovered during experimental studies. Fickett and Hatzigeorgiou [7] presented one of the first reviews of eukaryotic promoter prediction programs. Among these were oligonucleotide content-based neural network and linear discriminant approaches.

It was shown that many general-purpose promoter prediction programs can typically recognize only ~50% of the promoters with false positive (FP) rate of ~1 per 700–1000 bp [7]. The study to make a critical assessment of the human promoter prediction field also demonstrated a pretty low level of sensitivity of 58% for the specificity of 92% and correlation coefficient (CC) ranged from 0.52 to 0.73 for evaluated promoter predictors [8]. Much better accuracy has been observed for methods of identification of plant promoters [9–14]. Their specificity level, however, does not exceed 90% that will generate significant number of false positives when the methods would be applied to analyze long genomic sequences. The top two performers TSSP_TCM [9] and Promobot [10] with Sn = 0.88-0.89 and Sp = 0.84-0.86 outperform NNPP [11] (Sn/Sp:0.74/0.70), PromoterScan [12] (Sn/Sp:0.08/0.04), Promoter [13] (Sn/Sp:0.24/0.34), and Prom-Machine [14] (Sn/Sp:0.86/0.81).

While bacterial promoters have simpler structure than transcription initiation regions of higher organisms, their identification is also a challenging task. Using sequence alignment kernel and SVM classifier Gordon et al. [15] achieved Sn = 0.82 and Sp = 0.84 discriminating between *σ*70 promoter and non-promoter *E.coli* sequences. Similar accuracy was observed for popular bacterial promoter prediction program Bprom [16]. These programs clearly outperform the NNPP (trained on *E.coli* K12 sequences) [11] and SIDD [17] programs. For example, SIDD correctly predicted only 74.6% of actual promoters with a false positive rate of 18%. When NNPP correctly predicted 66.4% of the real promoters, its false positive rate was 22.4%.

Thousands bacteria and eukaryotic genomes are already sequenced and many more are on the way, while little transcriptional information is available for most of them. Moreover, new genomes may have promoter features that are different from those observed in model organisms. For example, recent studies have shown that TATA boxes and Initiators are not universal features of plant promoters, and that other motifs such as Y patches may play a major role in the transcription initiation in plants [10, 18, 19]. We face the situation that specific promoter characteristics that are often used in developing promoter predictors are poorly understood in many new genomes. This creates favorable circumstances for developing universally applicable algorithm of promoter prediction and in this paper we propose the use of convolutional neural networks, with an input consisting of only genomic sequence, as a rather general approach to solution of this problem.

Deep convolutional neural networks are capable of achieving record-breaking results in processing images, video, speech and audio on highly challenging datasets using purely supervised learning and they have recently won a large number of contests in pattern recognition and machine learning [20–23]. There are a few successful examples of applying them to biological problems. Deep learning–based algorithmic framework, DeepSEA, can predict chromatin effects of sequence alterations and prioritize functional SNPs by learning a regulatory sequence code from large-scale chromatin-profiling data [24]. Improved performance for this task was reported using DanQ [25], a hybrid framework that combines convolutional and bi-directional long short-term memory recurrent neural networks [26]. Chen et al. applied deep learning method (abbreviated as D-GEX) to infer the expression of target genes from the expression of landmark genes [27]. Finally, DeepBind, a computational approach based on deep convolutional neural networks, can discover new DNA and RNA binding sites using a set of sequences and, for each sequence, an experimentally determined binding score [28].

In this paper we utilize Convolutional Neural Networks (CNN) to analyze sequence characteristics of prokaryotic and eukaryotic promoters and build their predictive models. The developed CNN models, implemented in CNNProm program, demonstrated the ability of deep learning to grasp complex promoter sequence characteristics and achieve significantly higher accuracy compared to previously developed promoter prediction programs.

## Materials and methods

### Training and testing data

In this study, in order to demonstrate universality of the suggested approach to promoter prediction problem we selected promoter sequences from very distant groups of organisms: two bacteria, human, mouse and a plant. The studied number of promoter and non-promoter sequences for each organism is shown in Table 1.

**Table 1:** Numbers, lengths and locations of promoter and non-promoter sequences for studied organisms. Locations are given relative to the TSS (Transcription Start Site) position.

Organism	#promoter sequences	#non-promoter sequences	Length/Location
*Escherichia coli* s70	839	3000	81/-60 - +20
*Bacillus subtilis*	746	2000	81/-60 - +20
Human TATA	1426	8256	251/-200 - +50
Human non-TATA	19811	27731	251/-200 - +50
Mouse TATA	1255	3530	251/-200 - +50
Mouse non-TATA	16283	24822	251/-200 - +50
*Arabidopsis* TATA	1497	2879	251/-200 - +50
*Arabidopsis* non-TATA	5905	11459	251/-200 - +50

We used bacterial promoter and non-promoter sequences of length 81 nt (nucleotides). Bacterial non-promoter sequences were taken from the corresponding genome sequences: we randomly selected fragments of protein-coding genes and took their opposite (non-coding) chain sequences. *Escherichia coli* *σ*70 promoter sequences were extracted from manually curated RegulonDB [29]. *Bacillus subtilis* promoters were taken from a collection described in [30]. As for human, mouse and *Arabidopsis* non-promoter sequences (size 251 nt) we used random fragments of their genes located after first exons. Eukaryotic promoter sequences were extracted from the well-known EPD database [31].

We used 20% of each set sequences in our test sets. 70% of the remaining sequences were used as training and 10% as validation sets. Training sets provide data to generate parameters of CNNmodels, while validation sets are used to find the optimum number of learning epochs (cycles) that should be limited to avoid over-fitting.

### Convolutional networks

Convolutional layer is a core building block of convolutional networks [20–23]. A layer consists of filters, which are small matrices (*W*), for example *L* × *L* × *D*, where *D* is depth of input data and *L* is called filter length. These filters are convolved with an input, i.e. moved spatially across an input, and a dot product is calculated for each position: *W* × *x* + *b*, where *W* is our filter, *x* is a small chunk of an input and *b* is bias. A local *L* × *L* area in our input is called a receptive field, and a distance of each step of a filter sliding across an input is called stride. Calculating a dot product at each position gives us an activation map for our filter. Next layer takes as an input activation maps from all filters. Activation map is in fact partially connected neurons, which share the same weight, i.e. weight corresponding to a filter. This weight sharing is an important property of convolutional networks. It dramatically reduces a number of required parameters compared to a fully connected layer.

Convolutional layer can be followed by another convolutional layer, in which case the depth of the input is the number of filters from a previous layer. Convolutional layers are eventually followed by a pooling layer. This is a simple layer that operates on each activation map, making it smaller and more manageable. The most common pooling technique is Max-Pooling, which chooses the largest of several values for further representation. Convolutional layers augmented by Max-Pooling are prevalent in many modern Deep Learners [23]. They can be useful for working with biological sequences because convolution filters can capture information on functional sequence motifs.

### CNN architecture for building promoter recognition models

**Fig 1:**
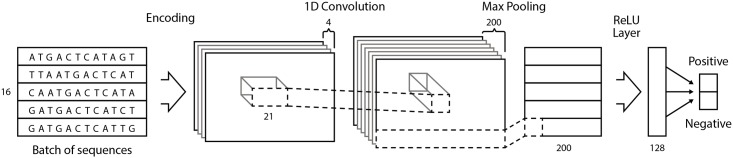
Basic CNN architecture that was used in building promoter models implemented in the learnCNN.py program (see text for description).

There are many network architectures and the task is to choose a suitable one for a particular research problem. In **learnCNN.py** program we implemented CNN model using Keras—a minimalist, highly modular neural networks library, written in Python [32]. It uses Theano library [33, 34] as a backend and utilizes GPU [35] for fast neural network training. Adam optimizer [36] was used for training with categorical cross-entropy as a loss function. Our CNN architecture (Fig 1) in most cases consisted of just one convolutional layer with 200 filters having length 21. After convolutional layer, we have a standard Max-Pooling layer. The output from the Max-Pooling layer is fed into a standard fully connected ReLU layer with 128 neurons. Pooling size was usually 2. Finally, the ReLU layer is connected to output layer with sigmoid activation, where neurons correspond to promoter and non-promoter classes. The batch size used for training was 16.

Input of the network consisted of nucleotide sequences where each nucleotide is encoded by a four dimensional vector A (1,0,0,0), T(0,1,0,0), G(0,0,1,0) and C(0,0,0,1). Output is a two dimensional vector: promoter (1, 0) and Non-promoter (0, 1) prediction. The training takes a few minutes on GTX 980 Ti GPU.

**Fig 2:**
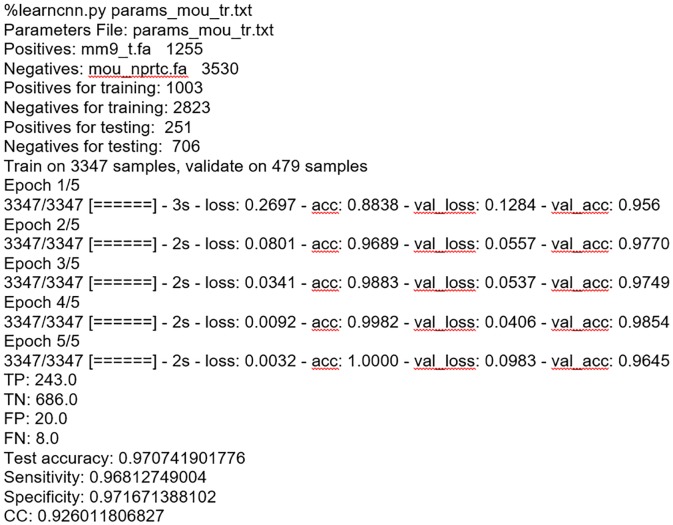
An example of learning CNN models for mouse promoters. The training, and validation accuracy is presented for each learning epoch. Finally, the performance on the test data is shown.

We intentionally used, in most cases, one layer CNN architecture, but sometimes to get a proper balance of accuracy between positives examples (promoters) and negative examples (non-promoter) two or three layers may be applied. A typical example of the model computation is shown in Fig 2.

### Performance measures

Several measures to estimate the accuracy of a recognition function were introduced in genomic research [37, 38]. Consider that we have *S* sites (positive examples) and *N* non-sites (negative examples). By applying a recognition function, we correctly identify TP sites (true positives) and TN non-sites (true negatives). At the same time FP (false positives) sites were wrongly classified as non-sites and FN (false negative) non-sites were wrongly classified as sites. Sensitivity (Sn) (true positive rate) measures a fraction of the true positive examples that are correctly predicted: *Sn* = *TP*/(*TP* + *FN*). Specificity (Sp) (true negative rate) measures a fraction of the predicted sites that are correct amongst those predicted: *Sp* = *TN*/(*TN* + *FP*). Accuracy *AC* = (*TP* + *TN*)/(*TN* + *TP* + *FN* + *FP*) measures an average performance on positive and negative datasets. However, this measures does not take into account the possible difference in sizes of site and non-sites sets. More correct single measure (correlation coefficient) takes the relation between correctly predictive positives and negatives as well as false positives and negatives into account [38]:

$$CC=\frac{TP\times TN-FP\times FN}{\sqrt{\left(TP+FP)(TP+FN)(TN+FP)(TN+FN\right)}}$$ (1)

## Results and discussion

### The accuracy of promoter identification by constructed CNN models

Using CNN architecture described above implemented in **learnCNN.py** program we analyzed the promoter and non-promoter sequences (Table 1). **LearnCNN.py** learns parameters of CNN model and outputs the accuracy of promoter prediction for the test set of sequences. It also writes computed CNN Model (PPCNNmodel) to a file, which can be used later in programs for promoter identification in a given sequence.

The accuracy information and some parameters of CNN architecture used for the particular datasets are show in Table 2.

**Table 2:** The accuracy and parameters of CNN models. Architecture as 200, 21, 4 describes one layer with 200 filters, filter length 21 and pooling size 4; ‘/’ separates data for two layers.

Organism	Sn	Sp	CC	CNN architecture
*Escherichia coli* s70	0.90	0.96	0.84	100,7, 0 / 150, 21, 12
*Bacillus subtilis*	0.91	0.95	0.86	100,15, 2 / 250, 17, 2
Human TATA	0.95	0.98	0.90	200, 21, 4
Human non-TATA	0.90	0.98	0.89	300, 21, 231
Mouse TATA	0.97	0.97	0.93	200, 21, 6
Mouse non-TATA	0.88	0.94	0.83	100, 15, 2 / 250, 21, 2
*Arabidopsis* TATA	0.95	0.97	0.91	200, 21, 4
*Arabidopsis* non TATA	0.94	0.94	0.86	200, 21, 2

We found that the computed CNN models demonstrated the ability of deep learning to grasp complex promoter sequence characteristics and achieve significantly higher accuracy compared to previously developed promoter prediction programs. For example, CNN trained on sigma70 sub-class of *Escherichia coli* promoters provides an excellent classification of promoters and non-promoter sequences (Sn = 0.90, Sp = 0.96). For human, mouse and *Arabidopsis* promoters, we employed CNNs to identify two well-known promoter classes (TATA and non-TATA promoters). CNN models nicely recognize these complex functional regions. For human, Sn/Sp accuracy of prediction reached 0.95/0.98 for TATA and 0.90/0.98 for non-TATA promoter sequences. For mouse, Sn/Sp accuracy of prediction achieved 0.97/0.97 for TATA and 0.88/0.94 for non-TATA promoters. The same outstanding performance was observed on *Arabidopsis* data: Sp/Sn for TATA promoters 0.95/0.97 and for non-TATA promoters 0.94/0.94. This is a very significant improvement in prediction performance compared to previously evaluated human promoter predictors, where the sensitivity of 58% and specificity of 92% with correlation coefficient (CC) in the range of 0.52–0.73 were observed [8].

In this work, we studied sequences of promoter regions extracted from the EPDnew promoter database [31], which recently extended promoter collection beyond cases based on evidence from TSS mapping experiments on single genes. Currently, TSS positions provided by EPD are inferred from next-generation sequencing data and are automatically generated from multiple, carefully selected input datasets that include chromatin signatures in addition to mRNA 5’tags to improve location of promoters for weekly expressed genes. The authors of EPDnew database have demonstrated its higher quality over ENSEMBL-derived [39] human promoter set [31]. We also observed apparent better quality of a promoter identification program when using EPDnew data. For example, for CNN predictor computed on 1083 mouse TATA promoter regions extracted from DBTSS [40], we also reached a pretty good performance on a test set of 271 promoters: Sn = 0.94, Sp = 0.94 and CC = 0.86. However, CNN model trained using mouse TATA promoters regions from EPDnew demonstrated noticeably better results: Sn = 0.94, Sp = 0.98 and CC = 0.93 (Table 2).

We would like to point out an important benefit of the considered CNN models. While using only nucleotide sequences, they can outperform recognition functions built based on preselected significant features. For example, widely used Bprom [16] promoter prediction program utilizes a set of seven features (five relatively conserved sequence motifs, represented by their weight matrices, the distance between −10 and −35 elements and the ratio of densities of octa-nucleotides overrepresented in known bacterial transcription factor binding sites relative to their occurrence in the coding regions). Computing these features for a set of 839 experimentally verified *σ*70 promoters from Regulon database [29] and 3000 non-promoter *E.coli* sequences and using LinearDiscriminantAnalysis and other discrimination approaches from scikit-learn Python library [41], we reached an average accuracy of 0.92 for classification of promoter and non-promoter sequences by applying cross-validation evaluation. The CNN model demonstrated a better recognition rate (Table 2) for the same data.

To apply our Promoter Prediction CNN (PPCNN) models to classifying sequences into promoters and non-promoters we designed **CNNprom.py** program. It takes the fasta format files as an input, together with the model parameters file, and outputs classification results for each sequence. If a sequence is classified as a promoter, the score assigned by network is provided in an output as well. To build the CNNProm program execution environment we installed Python, Theano and Keras libraries [32, 34] on the Softberry public Web server. The program can be run to analyze sequences of five studied organisms (two bacterial and three eukaryotic) at http://www.softberry.com/berry.phtml?topic=index&group=programs&subgroup=deeplearn. The developed PPCNN models have been recently applied in the programs for finding promoters in genomic sequences, which are also accessible at that server. The data sets with promoter and non-promoter sequences used to train and test CNN models can be downloaded at https://github.com/solovictor/CNNPromoterData.git.

### Random substitution method to discover positionally conserved functional elements

**Fig 3:**
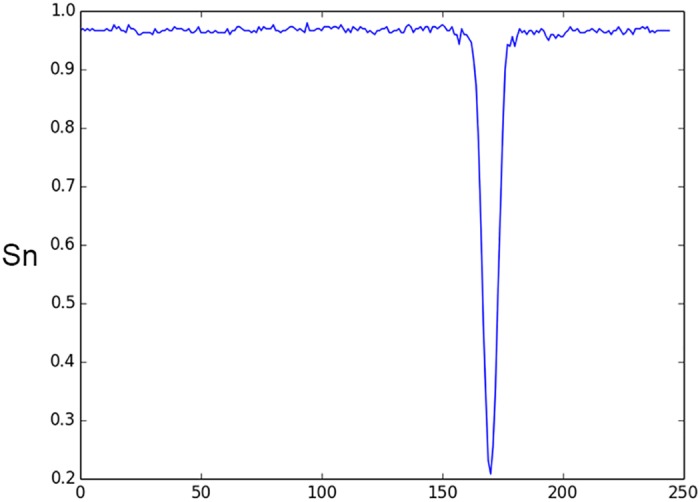
Effect of 6-nt sequence window substitution by random sequence on accuracy of classification of human TATA promoters. X-axis is the window position, Y-axis is promoter identification sensitivity after a substitution.

By analyzing network behavior we can extract some information on significant elements of the input data. Promoter sequences usually contain binding sites of regulatory proteins. Some of them occupy various locations relative to TSS and can be found in direct or complementary DNA chain. However, there are a number of well-known functional sites (such as bacterial -10 –box or eukaryotic TATA-box) that occupy approximately the same position in each promoter sequence. To discover such sites we suggest the following procedure. Take a window of length *L* (including positions from *x*_1_ to *x*_2_) and change the sequence within this window to a random sequence. Evaluate the accuracy of the site prediction after such change. Using sliding window moving from the beginning of a functional site sequence, we can build a performance profile that reflects an effect of a random sequence, inserted in each sequence position in place of an original sequence, on the accuracy of the site prediction. An example of such profile computed with window size 6 nt is shown in Fig 3.

**Fig 4:**
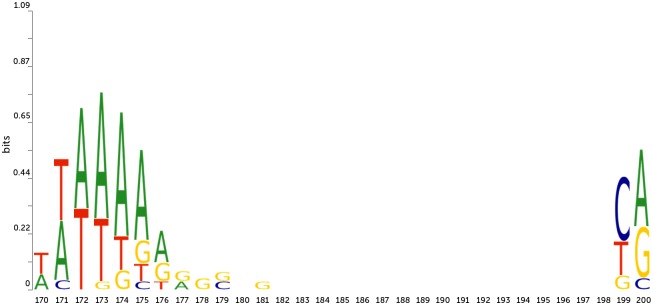
Sequence logo for human TATA promoter sequences in the TATA-box region and TSS region. X-axis is a position in promoter sequence, Y-axis is informational content in bits.

We can see that substitution of the sequence located between -45 and -20 positions relative to TSS (located in position 201) of human promoters drastically decreases the prediction accuracy. These positions include the well-known functional motif called TATA-box. The sequence logo [42] demonstrating conserved sequences of that motif is shown in Fig 4.

**Fig 5:**
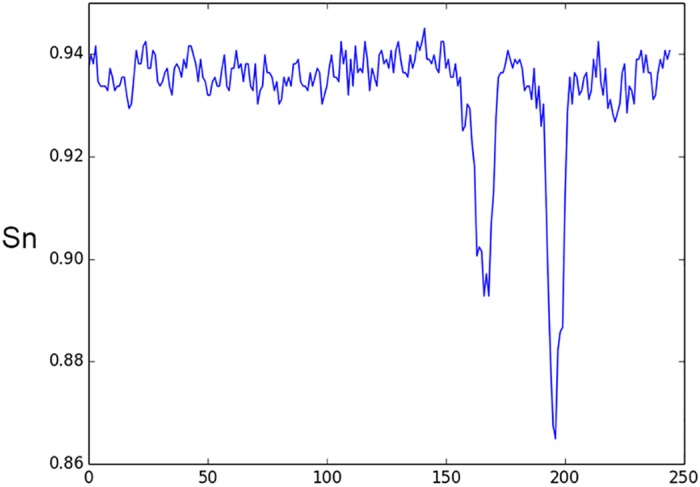
Effect of 6-nt sequence window substitution by random sequence on accuracy of classification of *Arabidopsis* non-TATA promoters. X-axis is a position of a window, Y-axis is promoter identification sensitivity after a substitution.

Another interesting example was observed while applying the random substitution procedure to *Arabidopsis* non-TATA promoters, see Fig 5.

**Fig 6:**
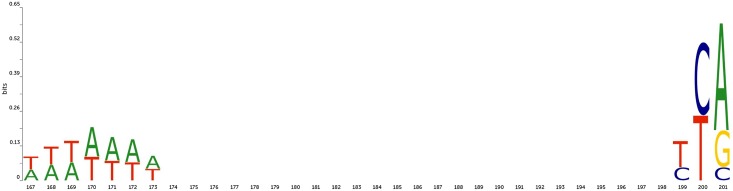
Sequence logo for *Arabidopsis* non-TATA promoter sequences. X-axis is a position in promoter sequence, Y-axis is informational content in bits.

Here we observe two positionally conserved and potentially functionally important elements (Fig 6). One is located approximately in positions -34–-28 and another in positions -2–0 relatively to TSS (located in position 201).

Thus, the suggested random substitution procedure can enable discovery of functionally important sites (sub-regions) that are still often unknown. Due to relatively high accuracy of CNNprom promoter prediction it would be interesting to use it in known or predicted upstream gene regions in combination with gene-recognition software tools to improve gene identification accuracy, as well as to annotate promoter regions.

## Conclusion

Present study demonstrates very good performance of CNN models in classifying promoter and non-promoter sequences. Accurate identification of promoters in long genome sequences, however, remains a major challenge, requiring not only accurate classifiers, but also appropriate selection of unique predictions among multiple overlapping high scoring genomic segments. In this task, it is also very important to account for multiple or alternative promoters for each transcription unit, possibly applying nonparametric methods recently described and tested on promoter regions of a model dicot plant *Arabidopsis thaliana* [43]. While we already incorporate developed CNN classifiers into a program of promoter identification in genome sequences, the approaches to resolve many difficult aspects of this task will be considered in our follow-up studies.

The suggested application of deep learning in promoter prediction and positional analysis of functional sites does not require knowledge of any specific promoter features. Since the convolution filters are able to automatically capture sequence motifs and other significant characteristics of biological/genomic sequences, this approach can be easily extended to identify promoters and other complex functional regions in sequences of many other genomes, making it very useful, especially considering that complete genomic sequence of thousand organisms will soon be available and how little transcriptional information is available for most of them.
